# Supplementary material for: Empirical methods for controlling false positives and estimating confidence in ChIP-Seq peaks
Source: BMC Bioinformatics. 2008 Dec 5;9:523. doi: 10.1186/1471-2105-9-523 (PMC2628906; doi:10.1186/1471-2105-9-523)
Supplement: Additional File 1 — A variety of html documents from the USeq web site detailing the available applications, their best usage, output file type descriptions, command line menus, etc. [file 1471-2105-9-523-S1.zip › USeqUserGuides/JavaDoc.zip!/JavaDoc/edu/utah/seq/data/HeatMapMaker.html]

HeatMapMaker


|  |  |  |
| --- | --- | --- |
| **Overview** \| **Package** \| **Class** \| **Use** \| **Tree** \| **Deprecated** \| **Index** \| **Help** | |  |
| **PREV CLASS**   **NEXT CLASS** | **FRAMES**    **NO FRAMES**     **All Classes** |  |
| SUMMARY: NESTED \| FIELD \| CONSTR \| METHOD | DETAIL: FIELD \| CONSTR \| METHOD |  |


---


## edu.utah.seq.data Class HeatMapMaker

```
java.lang.Object
  edu.utah.seq.data.HeatMapMaker
```

---

``` public class HeatMapMaker extends java.lang.Object ```

Converts a SmoothingWindow[] into a heat map specific bar file for import into IGB.
Assumes windows are sorted by chromosome and start position
Score index is the index to use when fetching a score for a window from it's double[] of scores.

---

**Constructor Summary**

`HeatMapMaker(int scoreIndex,
float baseScore)`


| **Method Summary** | |
| --- | --- |
| `boolean` | `baseOverlapsWindow(int bp, SmoothingWindow win)`             Is a given base contained within the window? Interbase coordinates so last window position is not included. |
| `float` | `getBaseScore()` |
| `int` | `getScoreIndex()` |
| `double` | `highestScoringWindow(int base, int currentWindowIndex)`             Looks forward at overlapping windows, returns highest score for the given bp position. |
| `PointData` | `makeHeatMapPositionValues(SmoothingWindow[] windows, boolean oneDecimal)`             Returns a PointData object with loaded position and values representing a top score heatmap/ stairstep. |
| `void` | `setBaseScore(float baseScore)` |
| `void` | `setScoreIndex(int scoreIndex)` |

**Methods inherited from class java.lang.Object**

`equals, getClass, hashCode, notify, notifyAll, toString, wait, wait, wait`

**Constructor Detail**

### HeatMapMaker

```
public HeatMapMaker(int scoreIndex,
                    float baseScore)
```


**Method Detail**

### makeHeatMapPositionValues

```
public PointData makeHeatMapPositionValues(SmoothingWindow[] windows,
                                           boolean oneDecimal)
```

:   Returns a PointData object with loaded position and values representing a top score heatmap/ stairstep.
    Be sure to expand the SmoothingWindows so their start and stops represent the true read size and NOT the
    middle position. Interbase coordinates.

---


### highestScoringWindow

```
public double highestScoringWindow(int base,
                                   int currentWindowIndex)
```

:   Looks forward at overlapping windows, returns highest score for the given bp position.

---


### baseOverlapsWindow

```
public boolean baseOverlapsWindow(int bp,
                                  SmoothingWindow win)
```

:   Is a given base contained within the window?
    Interbase coordinates so last window position is not included.

---


### getScoreIndex

```
public int getScoreIndex()
```

---


### setScoreIndex

```
public void setScoreIndex(int scoreIndex)
```

---


### getBaseScore

```
public float getBaseScore()
```

---


### setBaseScore

```
public void setBaseScore(float baseScore)
```


---


|  |  |  |
| --- | --- | --- |
| **Overview** \| **Package** \| **Class** \| **Use** \| **Tree** \| **Deprecated** \| **Index** \| **Help** | |  |
| **PREV CLASS**   **NEXT CLASS** | **FRAMES**    **NO FRAMES**     **All Classes** |  |
| SUMMARY: NESTED \| FIELD \| CONSTR \| METHOD | DETAIL: FIELD \| CONSTR \| METHOD |  |


---
